# Sperm-Storage Defects and Live Birth in *Drosophila* Females Lacking Spermathecal Secretory Cells

**DOI:** 10.1371/journal.pbio.1001192

**Published:** 2011-11-08

**Authors:** Sandra L. Schnakenberg, Wilfredo R. Matias, Mark L. Siegal

**Affiliations:** Department of Biology, Center for Genomics and Systems Biology, New York University, New York, New York, United States of America; Cornell University, United States of America

## Abstract

Male *Drosophila* flies secrete seminal-fluid proteins that mediate proper sperm storage and fertilization, and that induce changes in female behavior. Females also produce reproductive-tract secretions, yet their contributions to postmating physiology are poorly understood. Large secretory cells line the female's spermathecae, a pair of sperm-storage organs. We identified the regulatory regions controlling transcription of two genes exclusively expressed in these spermathecal secretory cells (SSC): *Spermathecal endopeptidase 1* (*Send1*), which is expressed in both unmated and mated females, and *Spermathecal endopeptidase 2* (*Send2*), which is induced by mating. We used these regulatory sequences to perform precise genetic ablations of the SSC at distinct time points relative to mating. We show that the SSC are required for recruiting sperm to the spermathecae, but not for retaining sperm there. The SSC also act at a distance in the reproductive tract, in that their ablation: (1) reduces sperm motility in the female's other sperm-storage organ, the seminal receptacle; and (2) causes ovoviviparity—the retention and internal development of fertilized eggs. These results establish the reproductive functions of the SSC, shed light on the evolution of live birth, and open new avenues for studying and manipulating female fertility in insects.

## Abstract 2

Transgenic *Drosophila* are used to identify the functions of a small set of secretory cells that are typically associated with the sperm-storage organs of female insects.

## Author Summary

Females of many animal species store sperm after mating, but the molecular and cellular mechanisms of sperm storage and maintenance are largely unknown. *D. melanogaster* females store sperm in the seminal receptacle and the paired spermathecae. Each spermathecal cap is lined with large secretory cells. There has been little direct evidence about the functions of these cells because we have not had the tools to manipulate the cells in otherwise wild-type females. Here, by creating transgenic tools to ablate the spermathecal secretory cells (SSCs) at different times relative to mating, without affecting any other cells, we show that SSCs are required to recruit sperm to the spermathecae but not to retain them there. We further show that SSC products act elsewhere in the reproductive tract in at least two ways. First, the SSCs are required to maintain sperm stored in the other storage organ, the seminal receptacle. Second, the SSCs are required to sustain normal egg laying. In the absence of SSCs, fertilized eggs develop and occasionally hatch as larvae inside the female. These results could have implications for understanding the evolution of sperm storage and live birth, as well as for studying and manipulating insect fertility.

## Introduction

Females of many animal species store sperm after mating, in specialized organs of the reproductive tract [1],[2]. In addition to sperm, seminal proteins are transferred to females during mating. In *Drosophila*, seminal proteins perform multiple functions that advance the male's reproductive interests. These functions include promoting sperm storage, decreasing the female's receptivity to subsequent courters, and stimulating egg production and ovulation [3],[4].

Female reproductive interests do not necessarily coincide with those of their mates. A coevolutionary arms race can therefore ensue [5]. In *Drosophila*, the coevolutionary pressure on female reproductive functions is apparently quite strong: seminal fluid decreases female lifespan [6] and does so to a greater extent when females are experimentally prevented from coevolving with males [7]. Of course, males and females do share some reproductive interests, such as successful production of offspring, and therefore evolutionary pressure also exists to coordinate their reproductive functions [8]. Although it has been appreciated that molecular interactions—both antagonistic and cooperative—between male and female products are key to understanding insect fertility and its evolution, much more progress has been made in characterizing the composition and functions of seminal fluid [3] than in characterizing female reproductive secretions.

A potentially major role of female secretions is in sperm storage. Insect females typically have multiple specialized sperm storage organs, to which sperm are recruited after copulation and in which sperm can be maintained for weeks or, in the case of queens of social insect species, years [9]. Female *Drosophila melanogaster* have three such organs located at the anterior of the uterus: a long tubular seminal receptacle and a pair of spermathecae. Each spermatheca is mushroom shaped, with a duct that extends from the uterus to a cuticular cap. The seminal receptacle houses up to 80% of stored sperm, whereas the spermathecal caps house the remainder [1],[10]. Each spermathecal cap is lined with large glandular cells containing prominent secretory organelles that open into the lumen where sperm are stored [11],[12]. Despite considerable divergence in sperm-storage organ anatomy, such cells are found lining or adjacent to the spermathecae of a wide range of insects [9]. The position of these spermathecal secretory cells (SSC) suggested they might have a role in sperm storage, yet direct in vivo evidence has been lacking [9],[12],[13]. Indeed, the most direct evidence for a role in sperm storage comes from a 1975 study of boll weevils with surgically removed spermathecal glands: sperm did not enter the spermatheca in such females, although because sperm motility was greatly diminished it cannot be concluded whether the glands are necessary just for sperm viability or also for recruitment into storage [14].

The SSC might also contribute to sustained levels of egg production and fertilization, by secreting proteins that alter female reproductive physiology or by modulating the activities of male seminal proteins. Sex peptide, a seminal protein, binds to sperm tails and during storage is gradually cleaved to an active form that stimulates egg production [15]. Sex peptide is also required for release of sperm from storage [16]. In the female, the sex peptide receptor is expressed in neurons that mediate a decrease in courtship receptivity and an increase in egg laying after mating, and it is required for these changes [17]. The sex peptide receptor is also expressed in the SSC [17], suggesting that sex peptide acts directly on these cells.

Other genes expressed specifically in the SSC have not been comprehensively identified, although some such genes have emerged from transcriptional profiling studies of: (1) somatic tissues of males versus females [18], (2) dissected whole spermathecae [11],[19],[20], and (3) virgin versus mated females [21]–[23]. One notable class of genes revealed by these studies is those encoding proteases. Protease-encoding genes are over-represented among those induced in females by mating [21]–[23], and among those highly expressed in the spermathecae [11],[19],[20]. Proteases are especially interesting due to their potential for interactions with seminal proteins. Such interactions could be antagonistic, for example by degradation of seminal proteins, or cooperative, for example by regulated cleavage of seminal proteins to their active forms [8]. Male-female coevolution would be expected to lead to rapid divergence of female-expressed protease-encoding genes, and indeed such genes show elevated rates of coding-sequence evolution in several species [19],[24]–[30].

To address how the SSC contribute to female postmating physiology, we have developed tools that allow us to manipulate these cells in a precise spatiotemporal manner. To develop these tools, we first identified the regulatory regions controlling transcription of two protease-encoding genes that are expressed exclusively in the SSC. The gene *CG17012*, which encodes a serine-type endopeptidase, is expressed exclusively in the SSC, in both unmated and mated females [18]. We hereafter refer to *CG17012* as *Spermathecal endopeptidase 1* (*Send1*). *CG18125*, which also encodes a serine-type endopeptidase, is expressed exclusively in the SSC and its transcription is upregulated 76-fold 3–6 h after mating [27]. We hereafter refer to *CG18125* as *Spermathecal endopeptidase 2* (*Send2*). Identifying these genes' regulatory regions allowed us to create drivers and reporters for manipulating and monitoring the SSC.

We show that the SSC are required to recruit sperm to the spermathecae, but not for maintaining them there. Moreover, we show that the SSC act at a distance in the reproductive tract, in that they are required for maintaining sperm motility in the seminal receptacle. We also show action at a distance with respect to egg laying, in that females lacking SSC are ovoviviparous. Fertilized eggs develop, and indeed sometimes hatch into larvae, inside the uterus. This phenotype is reminiscent of two species of *Drosophila* that retain developing eggs, *D. sechellia* and *D. yakuba* [31]. Our results therefore not only reveal the functions of a poorly understood reproductive tissue, but also shed light on the evolution of live birth.

## Results/Discussion

### Construction of SSC-Expressed Driver and Reporter Genes

For each of the SSC-expressed genes, *Send1* and *Send2*, we created a driver carrying ∼4 kb of upstream sequence and ∼4 kb of downstream sequence, flanking the coding sequence of the yeast transcriptional activator GAL4, which can activate transgene expression in *Drosophila* through its cognate UAS sequence [32]. We also created GAL4-independent reporters by cloning a subfragment of the *Send1* or *Send2* upstream sequence in front of the coding sequence of a fast-maturing, nuclear-localized, red-fluorescent protein (DsRed.T4.NLS, hereafter called nRFP) [33].

**Figure 1:**
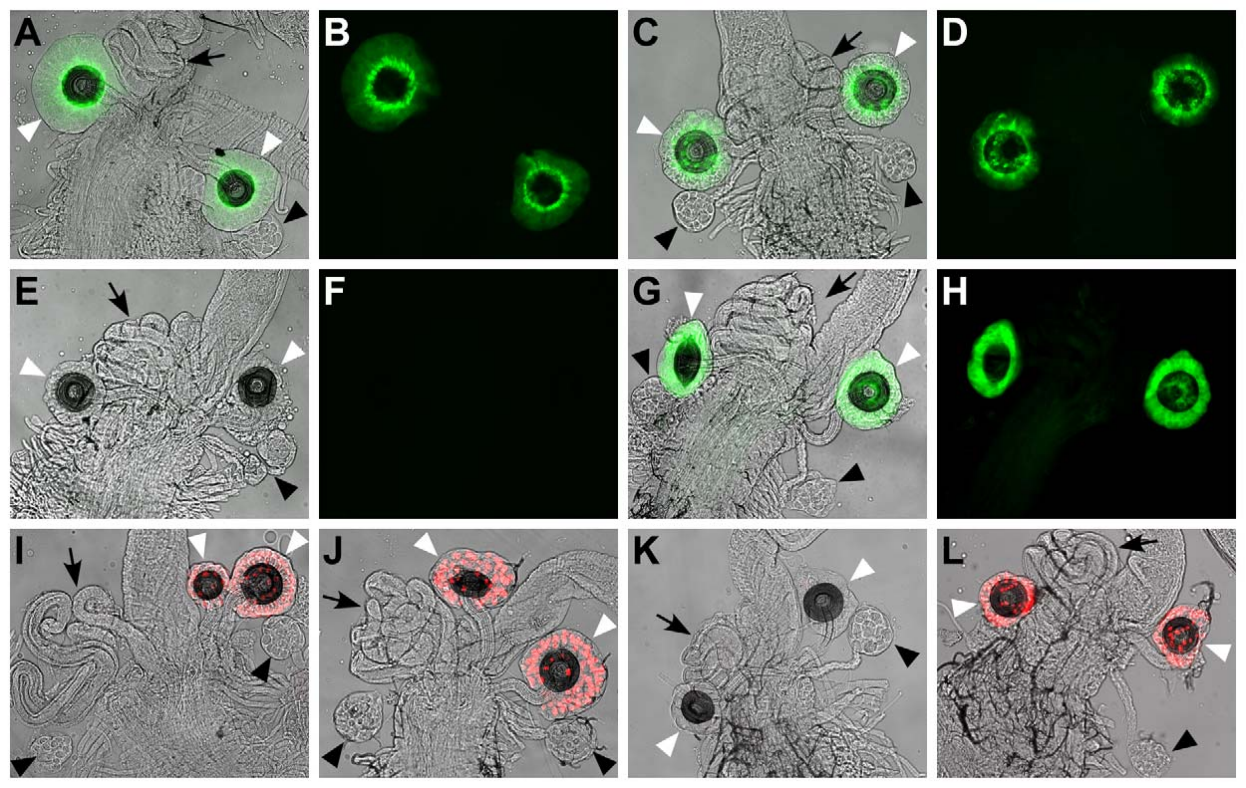
Activities of *Send1* and *Send2* GAL4 drivers and nRFP reporters in the lower reproductive tract. Virgin female with *Send1-GAL4* driving *UAS-mCD8-GFP*, shown as overlay of brightfield and GFP images (A) and as GFP alone (B). Mated female with *Send1-GAL4* driving *UAS-mCD8-GFP*, as overlay (C) and GFP alone (D). Virgin female with *Send2-GAL4* driving *UAS-mCD8-GFP*, as overlay (E) and GFP alone (F). Mated female with *Send2-GAL4* driving *UAS-mCD8-GFP*, as overlay (G) and GFP alone (H). Virgin (I) and mated (J) females with *Send1*-*nRFP*. Virgin (K) and mated (L) females with *Send2*-*nRFP*. White arrowheads indicate spermathecae; black arrowheads indicate accessory glands; and black arrows indicate seminal receptacles.

*Send1-GAL4* drives expression of a membrane-bound green fluorescent protein (GFP) (*UAS-mCD8-GFP*) specifically in the SSC of virgin and mated females (Figure 1A–1D). GFP expression driven by *Send1-GAL4* is visible by 20 h posteclosion and increases in intensity by day 4. *Send2-GAL4* drives GFP expression in the SSC of mated females only, as early as 3 h postmating (Figure 1E–1H). *Send1-nRFP* and *Send2-nRFP* recapitulate expression of the respective endogenous genes as well (Figure 1I–1K).

### Precise Ablation of the SSC before and after Mating

The potential for redundancy among spermathecae-expressed serine proteases is high. Protease-encoding genes are over-represented among those induced in females by mating [21]–[23], and among those highly expressed in the spermathecae [11],[19],[20]. Moreover, some spermathecae-expressed serine proteases are recently duplicated paralogs with high levels of amino acid identity. Consistent with redundancy, we did not observe any effects on female fecundity or fertility when we used *Send1-GAL4* to drive RNAi efficiently targeting *Send1* or *Send2* transcripts (see Materials and Methods).

To fully understand how SSC-expressed proteases contribute to female reproductive function might require the simultaneous knockdown or knockout of many genes. As an alternative approach, we used the *Send1-GAL4* and *Send2-GAL4* drivers to ablate the SSC at different times, thereafter eliminating their ability to secrete any products into the spermathecal lumen. As the SSC are terminally differentiated adult cells, we used the drivers to express a modified form of the apoptosis-promoting protein Hid (Hid^Ala5^) that is effective in postmitotic cells [34].

We assayed the timing and effectiveness of cell-death induction using the nRFP reporters, as well as direct visualization of apoptotic cells by TUNEL (Materials and Methods). For all assays with *Send1-GAL4*, we mated females on day 4 posteclosion, to ensure that the majority of their SSC had been ablated. Note that occasionally a few SSC remain at day 4. We use this lack of complete penetrance to our advantage because it creates some mosaic females who have one of the two spermathecae lacking SSC whereas the other still has SSC. With *Send2-GAL4*, the SSC are clearly apoptotic as early as 31 h postmating. By 43 h postmating, the majority of SSC are dead (Figure S1).

### Products of the SSC Are Required for Initial Storage of Sperm

**Figure 2:**
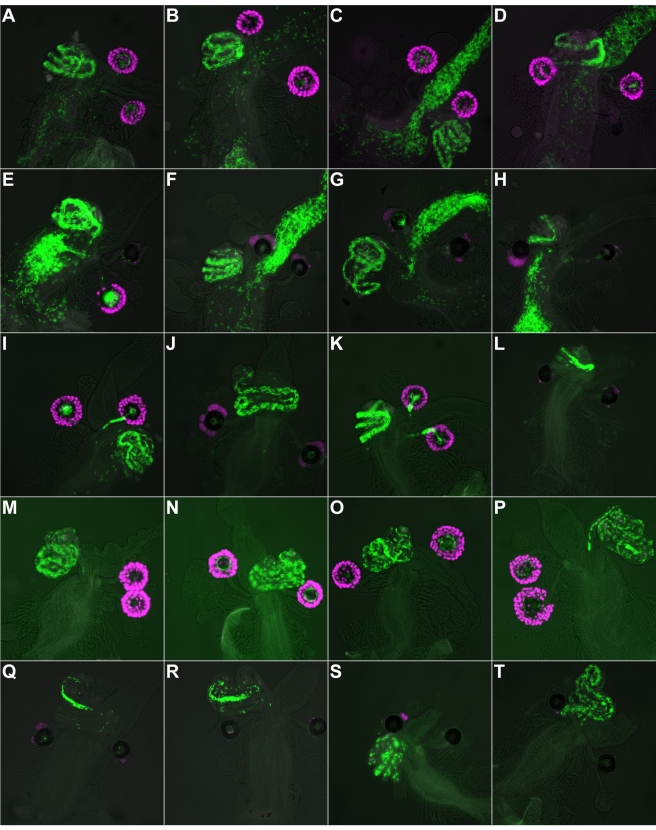
Sperm recruitment and maintenance defects in females with SSC ablated prior to mating. (A–D) Control females 1 h postmating. (E–H) SSC-ablated females 1 h postmating. (I) Control female 7 h postmating. (J) SSC-ablated female 7 h postmating. (K) Control female 24 h postmating. (L) SSC-ablated female 24 h postmating. (M–P) Control females 6–9 d postmating. (Q–T) SSC-ablated females 6–9 d postmating. SSC are magenta (*Send1-nRFP*) and sperm are green (*protamine-GFP*).

We examined sperm storage in SSC-ablated and control females by individually mating them with males expressing protamine-GFP, which renders sperm heads fluorescent green [35]. Males transfer between 3,000 and 4,000 sperm during mating, of which only ∼25% are stored. Of the stored sperm, approximately 65% to 80% reside in the seminal receptacle and the rest reside in the spermathecae [1],[10]. Mortality of stored sperm remains quite low for about 2 wk [36]. In control *+/UAS-hid^Ala5^; Sp/+; Send1-nRFP/+* females, sperm are stored in the spermathecae and seminal receptacle within 1 h of mating, although sperm are also found in the uterus and occasionally the oviduct (Figure 2A–2D). In their *+/UAS-hid^Ala5^; CyO, Send1-GAL4/+; Send1-nRFP/+* sisters, whose SSC were ablated prior to mating, sperm are also found in the seminal receptacle and uterus, and occasionally in the oviduct, but often are not present in the spermathecae (Figure 2E–2H). Indeed, out of 17 SSC-ablated females, 16 had at least one empty spermatheca, and eight of these had both spermathecae empty. By contrast, no control female had even one empty spermatheca, and in only one case did one of the spermathecae contain fewer than ten sperm (Table S1). In cases of SSC-ablated females in which sperm were found in one of the spermathecae but not the other, the presence or absence of sperm correlated with the presence or absence of SSC in a mosaic female (e.g., Figure 2E). These results imply that the SSC are required to recruit sperm to the spermathecae.

It was previously shown that glucose dehydrogenase, which is secreted from the proximal and distal ends of the spermathecal duct, promotes recruitment of sperm into the spermathecae [37]. We observed some cases in which sperm were present in the spermathecal duct leading to a spermathecal cap with no sperm (e.g., Figure 2H), but in most cases the ducts were empty of sperm as well. This result suggests that recruitment of sperm by the duct cells is largely dependent on SSC function.

By 7 h postmating, nearly all remaining sperm in control females are in the seminal receptacle or spermathecae (Figure 2I), as expected. By contrast, SSC-ablated females have sperm in their seminal receptacles, yet tend to lack sperm in the spermathecae (Figure 2J; Table S1). The lack of sperm in the spermathecae at 7 h suggests that sperm recruitment to the spermathecae is indeed impaired and not merely delayed.

### Products of the SSC Are Required to Maintain Sperm Stored in the Seminal Receptacle

At 24 h postmating, sperm storage in control females appears very similar to that observed at 7 h postmating. In SSC-ablated females, however, sperm dynamics in the seminal receptacle are aberrant. Whereas in control females, sperm are found throughout the tubular receptacle (Figure 2K), in many SSC-ablated females, sperm have lost motility and clumped together in one part of the receptacle, leaving the rest of it largely empty (Figure 2L; Table S1; Videos S1 and S2). This result suggests that the products of the SSC travel to, and act in, the seminal receptacle. Consistent with this inference, Anderson [38] found that females lacking entirely the spermathecae and female accessory glands lose fertility within a few days after mating. The loss of fertility is apparently caused by loss of motility of sperm stored in the females' seminal receptacles [38]. A similar conclusion was reached by Allen and Spradling [11], who used a different mutation that causes loss of the spermathecae and accessory glands. Our results localize the source of at least one motility-maintaining factor to the SSC.

At 6 to 9 d postmating, sperm storage in control females still appears very similar to that observed at 7 h or 24 h postmating (Figure 2M–2P; Table S1). Likewise, sperm storage at 6 to 9 d postmating in SSC-ablated females appears very similar to that observed in SSC-ablated females at 24 h postmating (Figure 2Q–2T). Although rare females contain a few sperm in their spermathecae (e.g., Figure 2Q), most do not (e.g., Figure 2R–2T). As at 24 h postmating, clumps of sperm in the seminal receptacle are also seen in some females (e.g., Figure 2Q–2R; Table S1).

### Loss of the SSC after Mating Does Not Reduce Long-Term Sperm Retention or Maintenance

The existence of a few sperm in the spermathecae of some SSC-ablated females approximately 1 wk postmating suggests that the SSC are not required to retain sperm in the spermathecae once they have been stored there. However, as described above, there is a correlation between sperm recruitment to the spermathecae and the existence of residual, nonablated SSC. It could be that the same residual SSC function that recruited the sperm to the spermathecae is sufficient to retain them there. To test definitively whether the SSC are required to retain sperm in the spermathecae, we eliminated the SSC after sperm were stored in the spermathecae. We did so by ablating the SSC after mating using *Send2-GAL4* in combination with *UAS-hid^Ala5^*.

**Figure 3:**
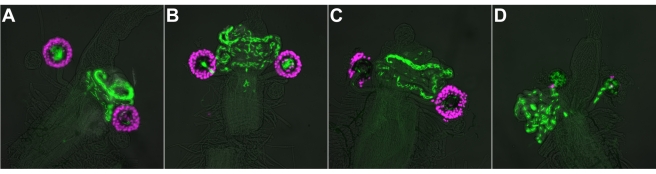
Sperm storage in females with SSC ablated after mating. (A) Control female 7 h postmating. (B) Experimental female 7 h postmating. (C) Control female 8 d postmating. (D) Experimental female 8 d postmating. SSC are magenta (*Send1-nRFP*) and sperm are green (*protamine-GFP*).

At 7 h postmating, sperm storage in control *+/UAS-hid^Ala5^; Send1-nRFP/+; MKRS/+* females does not appear different to that of their *+/UAS-hid^Ala5^; Send1-nRFP/+; Send2-GAL4/+* sisters (Figure 3A–3B). As noted above, SSC ablation is complete in the latter females within one more day. If the SSC are required for long-term sperm retention in the spermathecae, then SSC-ablated females should lose the sperm they had stored in the spermathecae. However, this is not the case. At 6–8 d postmating, SSC-ablated females retain as many sperm in their spermathecae as do their control sisters (Figure 3C–3D). Moreover, sperm clumping in the seminal receptacle is not observed in these SSC-ablated females, implying that whatever SSC products are required to maintain sperm motility need only be supplied up to or around the time of mating, not continually.

### Products of the SSC Are Required to Sustain Egg Laying

**Figure 4:**
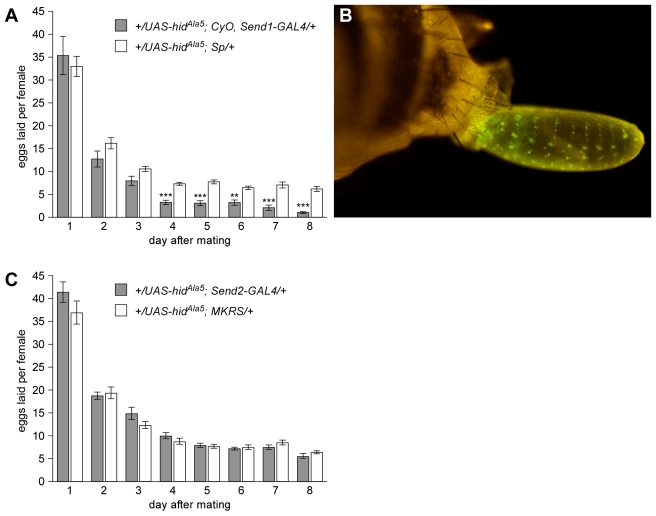
Reduced egg laying and ovoviviparity in females with SSC ablated prior to mating. (A) Number of eggs laid per female for each of days 1–8 postmating. Genotypes of experimental females with SSC ablated prior to mating (gray) and their control sisters (white) are as shown, plus each is heterozygous for the *Send1-nRFP* transgene. Error bars are standard error of the mean. Significant differences between experimental and control on a given day are as shown (**, *p*<0.01; ***, *p*<0.001). The entire distribution of egg counts across days is also significantly different between experimentals and controls (Kolmogorov-Smirnov test, *p*<0.05). (B) Late-stage embryo released from female with SSC ablated prior to mating. Cholinergic neurons of the embryo are labeled with *Cha*-*GAL4*, *UAS*-*GFP*, which is expressed starting at stage 16. (C) Number of eggs laid per female for each of days 1–8 postmating. Genotypes of experimental females with SSC ablated after mating (gray) and their control sisters (white) are as shown, plus each is heterozygous for the *Send1-nRFP* transgene. The distributions are not significantly different (Kolmogorov-Smirnov test, *p* = 1.00).

Because females whose SSC are ablated prior to mating do not store sperm in their spermathecae and lose sperm motility in their seminal receptacles, we next asked whether this impaired sperm storage affects fecundity or fertility. Females with SSC ablated prior to mating lay as many eggs on days 1 to 3 postmating as their control sisters (Figure 4A). However, after day 3, their egg laying is significantly reduced (Figure 4A). Notably, after day 3 an individual SSC-ablated female tends to lay vastly different numbers of eggs on successive days. Indeed, 10 out of 18 SSC-ablated females had one day in which 0 or 1 egg was laid, followed immediately by a day with greater than ten eggs laid. By contrast, only one out of 15 control females had any day in which 0 or 1 was laid.

The alternation of low and normal levels of egg laying in SSC-ablated females suggests that the SSC play some role in either ovulation or oviposition. To determine which is the case, we dissected SSC-ablated females that had not laid an egg in the previous 24 h. Strikingly, SSC-ablated females are ovoviviparous: a large proportion of such females (eight out of 16) had a late-stage embryo or live first-instar larva stuck in the uterus (Figure 4B; Video S3). This result implies a defect in ejecting eggs from the uterus (oviposition) rather than egg production and release (ovulation). However, those females with stuck eggs did not appear to have a “log jam” of eggs in the oviduct, suggesting either: (1) that inability to eject a fertilized egg signals back to the ovary to halt or slow ovulation [39],[40]; or (2) that ovulation is independently slowed by SSC loss.

A simple, mechanical explanation for the stuck-egg phenotype is that the SSC produce a lubricant that coats the uterus, allowing eggs to pass easily. An alternative explanation is that products of the SSC are required before or around the time of mating to trigger cellular and physiological changes in the reproductive tract that are required for full reproductive maturity. Consistent with the latter explanation, females whose SSC have been ablated postmating, using *Send2-Gal4*, do not show any difference from control sisters in the number of eggs laid on each of days 1 to 8 postmating (Figure 4C). This result implies that proper egg laying after day 3 postmating requires SSC function earlier in adulthood. The earlier function could be the production of a secretion, such as a lubricant, that is long-lived. However, multiple lines of evidence support the existence of posteclosion and postmating developmental programs by which the female reproductive tract achieves full maturity [41],[42]. The triggering of such a program by one or more SSC gene products could explain not only the egg-laying defect but also the loss of sperm motility in the seminal receptacle in females with *Send1*-driven, but not *Send2*-driven, ablation of the SSC.

### Conclusions

Work prior to ours had suggested several possible functions for the secretory cells of the spermathecae, including recruitment and maintenance of sperm, yet direct *in vivo* evidence was lacking because of the absence of tools for precisely manipulating these cells. In *D. melanogaster*, it had been observed that females lacking the spermathecae and accessory glands lose fertility, despite having normal seminal receptacles, but this effect could not be ascribed to any particular cell population within the missing organs [11],[38]. Our cell-specific drivers enabled us to determine that the SSC contribute to reproductive function in multiple ways, some expected and some not. The SSC do indeed produce one or more products required for recruiting sperm into storage, although they are not required to maintain sperm in the spermathecae once recruited. In contrast, the SSC are not required for sperm to reach the seminal receptacle, but they are required to maintain sperm motility there, consistent with the lost fertility of females lacking spermathecae and accessory glands [11],[38]. In addition to their action at a distance in the seminal receptacle, the SSC also act at a distance in sustaining egg laying. The impairment of egg laying in SSC-ablated females manifests as an unanticipated phenotype: ovoviviparity. SSC-ablated females retain fertilized eggs, which develop inside the uterus and, in some cases, hatch as larvae inside the mother.

Our findings have relevance to two evolutionary patterns observed in the genus *Drosophila*. First, all *Drosophila* species that have been examined have a pair of spermathecae and a single seminal receptacle, yet there are at least 13 independent *Drosophila* lineages in which females use only the seminal receptacle to store sperm [43]. In species that do not store sperm in the spermathecae, the spermathecal caps are small and weakly sclerotized, but are surrounded by large cells that are presumably their SSC [43]. Our finding that the SSC act at a distance in the female reproductive tract might explain why these species retain their spermathecae, despite not using them to store sperm.

Second, the ovoviviparity observed in SSC-ablated females suggests that transitions to live birth might require fewer evolutionary steps than once thought. In a surprising recent discovery it was found that two species of *Drosophila* are ovoviviparous: even when exposed to ample substrate for oviposition, females of *D. sechellia* and *D. yakuba* retain fertilized eggs, which develop internally, in contrast to those of all other examined *Drosophila* species, which are laid immediately after fertilization [31]. Our SSC-ablation results suggest that the *Drosophila* uterus is “preadapted” to support internal embryo development, in that eggs stuck there are capable of hatching into perfectly viable larvae.

The genetic tools we have developed will be useful for further dissection of the molecular and evolutionary mechanisms underlying female reproductive function. Insect reproductive secretions are of particular interest because of their relevance to the fertility of agricultural pests and human disease vectors. To date, attention has focused on male secretions [44]–[47], because of the far greater knowledge of male seminal proteins than of products of the female reproductive tract, but recent studies in malaria-vector mosquitoes have begun to counter this bias [48]. Reproductive secretions are also highly relevant to the study of beneficial insects, as evidenced by recent work characterizing the seminal-fluid and spermathecal-fluid proteomes of honey bees [49]–[51]. Increased knowledge of the regulation and functions of spermathecal secretions will add a new dimension both to insect-control efforts and to the maintenance of healthy breeding populations of agriculturally important insects.

## Materials and Methods

### Drosophila Stocks and Fly Husbandry

*P{w^+mc^ = UAS-mCD8::GFP.L}LL5* (referred to as *UAS-mCD8-GFP* in the text) and *w; P{w^+mC^ = Cha-GAL4.7.4}19B P{w^+mC^ = UAS-GFP.S65T}T2* (referred to as *Cha-GAL4, UAS-GFP* in the text) fly lines were obtained from the Bloomington Drosophila Stock Center. The Canton-S fly line was obtained from Michelle Arbeitman [52]. The *protamine-GFP* fly line was obtained from John Belote and Scott Pitnick. The *UAS-hid^Ala5^* fly line was obtained from Hermann Steller. Virgin flies were collected within 6 h of eclosion and maintained in single-sex groups for 4 to 5 d before mating. All virgin female flies were mated with Canton-S, *protamine-GFP* or *Cha-GAL4, UAS-GFP* males, in single pairs. Mated females were used only if copulation lasted at least 15 min. All flies were maintained on standard yeast-cornmeal-molasses medium at 25°C.

### Generation of GAL4 Drivers and nRFP Reporter Lines

The *Send1-GAL4* construct contains ∼8 kb of genomic sequence surrounding *Send1* (coordinates 2,249,863–2,258,703 on Chromosome 2L), with the *Send1* coding sequence precisely replaced with the coding sequence of GAL4. *Send2-GAL4* was similarly constructed and contains ∼8 kb of genomic sequence surrounding *Send2* (coordinates 14,341,694–14,350,575 on Chromosome 2L). Each *Send-GAL4* cassette was inserted separately into the *P*-element transformation vector pP{whiteOut2}, which was provided by Jeff Sekelsky. Reporter constructs were made by PCR-amplifying genomic fragments from *Send1* (coordinates 2,255,054–2,255,684) and *Send2* (coordinates 14,346,671–14,347,347) and cloning each separately into pRed H-Stinger, which places the cloned fragment upstream of a minimal promoter and the coding sequence for DsRed.T4.NLS [33]. Germline transformation of *w^1118^* embryos was carried out by Rainbow Transgenics.

### Transgenic RNAi Targeting *Send1* and *Send2*

RNAi-producing UAS-hairpin constructs were made for *Send1* and *Send2* by inserting inverted repeats into the *P*-element transformation vector pWIZ, a derivative of pUAST that separates the repeats by a small intron to facilitate cloning and enhance silencing [53]. For *Send1*, a 518-bp fragment starting at position 291 of the coding sequence was amplified by PCR, using primers with added *Spe*I restriction sites at their 5′ ends. The *Spe*I-digested PCR product was cloned into the *Nhe*I site of pWIZ and then again in inverted (head-to-head) orientation into the *Avr*II site of the resulting plasmid. For *Send2*, the cloning steps were identical to those for *Send1*, except the amplified fragment comprised the 514 bp starting at position 1 of the coding sequence. *P* elements were transformed into flies by standard methods [54],[55].

To test if loss of either the *Send1* or *Send2* transcript has an effect on female fecundity or fertility, we used the *Send1-GAL4* driver in combination with the *UAS-RNAi* construct as well as a *UAS-Dicer-2* transgene, which increases RNAi pathway activity [56], and a *UAS-GAL4* transgene, which sets up a positive-feedback loop of GAL4 expression [57]. *UAS-Dicer-2*; *CyO, Send1*-*GAL4*/*Sp*; *Send1*-*nRFP* males were mated to *+; UAS-Send1RNAi*; *UAS-GAL4* or *+; UAS-Send2RNAi*; *UAS-GAL4* females. Female progeny that did not inherit the *GAL4* driver (i.e., inherit the *Sp* chromosome) served as controls. 4-d-old virgin experimental and control females were mated individually to Canton-S males and incubated for 3 h at 29°C. Females were then shifted to 25°C in groups of five and transferred to fresh food vials every 24 h. The number of eggs laid was counted each day for 10 d postmating, as was the number of adult progeny produced from these eggs. The knockdown of each gene had no impact on fecundity relative to controls, as determined by Kolmogorov-Smirnov tests on the egg-count data (*p* = 0.313 for *Send1RNAi*, *p* = 0.675 for *Send2RNAi*). Likewise, the knockdown of each gene had no impact on fertility, as all eggs produced viable adults. We did confirm, however, that RNAi effectively targeted each gene's transcript. As assayed by quantitative RT-PCR with a *Gadph2* internal standard, *Send1* transcript levels in virgin and mated females were reduced to 10.2% and 9.4% of wild-type levels, respectively; *Send2* transcript levels in mated females were reduced to 1.5% of wild-type levels.

### Efficacy of SSC Ablation

The mutant Hid^Ala5^ cannot be phosphorylated by MAP kinase, which promotes survival of differentiated cells [34]. To test the efficacy of *Send1*-driven Hid^Ala5^ to trigger cell death, we mated *+/UAS-hid^Ala5^; CyO, Send1-GAL4/+; Send2-nRFP/+* females to males at day 2, 3, or 4 posteclosion. As assayed by activation of the *Send2-nRFP* reporter, the majority of SSC respond to mating when it occurs on day 2 posteclosion, but fail to respond to mating on or after day 3 posteclosion. To examine *Send2*-driven cell death, we used the TUNEL assay. Experimental *+*/*UAS*-*hid^Ala5^*; *+*/*Send1-nRFP*; +/*Send2*-*GAL4* females were aged for 4 d prior to mating with Canton-S males. Females were incubated at 29°C for 3 h postmating and transferred to 25°C for 21, 28, or 40 h. Lower reproductive tracts, including the spermathecae, were dissected in cold PBS with 0.05% Tween-20 and fixed in 2% paraformaldehyde, PBS, 0.05% Tween-20 for 1 h at 25°C. Tissues were then permeabilized for 5 min in a 1% Triton-X, 0.1% sodium citrate buffer at room temperature. Reproductive tracts were incubated with the TUNEL reagent (Roche) for 2 h at 37°C then washed in PBS, fixed for 30 min in 4% paraformaldehyde, mounted in Vectashield (Vector Labs) and maintained at 4°C.

### Sperm Visualization

After mating to *protamine-GFP* males, experimental and control females were incubated at 29°C for 3 h then shifted to 25°C. Ovaries and reproductive tracts of females were dissected at room temperature in Grace's medium (Fisher Scientific). Reproductive tracts were quickly removed and observed for 5 min to monitor sperm motility and score sperm clumping, using a Leica MZ16FA stereomicroscope with Plan Apo 2.0X objective.

### Sperm Dynamics

Female reproductive tracts were dissected in room-temperature Grace's medium and mounted in 35-mm glass-bottomed dishes (MatTek). Videos were collected at ten frames per second by spinning-disk confocal imaging on a Leica DM IRE2 inverted microscope with HC PL Apo 10×/0.40 CS objective. A single confocal plane was imaged for the duration of the video. The microscope was interfaced with a computer running Velocity v5.0.2 imaging software and a Hamamatsu EM-CCD digital camera.

### Visualization of Embryos from Ovoviviparous Females

To stage embryos, we mated *+/UAS-hid^Ala5^; CyO, Send1-GAL4/+; Send1-nRFP/+* females to homozygous *Cha-GAL4, UAS-GFP* males. *Cha-GAL4* contains 7.4 kb of 5′-flanking sequence of the *Choline acetyltransferase* (*Cha*) gene and is expressed in the brain, ventral nerve cord, and peripheral nervous system, starting at embryonic stage 16 [58]. Females that had not laid an egg for 24 h were gently anesthetized by cooling to allow for reflex oviposition and mounted in 100% silicone to stabilize the abdomen. Videos and still images of embryos were collected using a Leica MZ16FA stereomicroscope with Plan Apo 2.0× objective.

### Microscopy

All internal reproductive tract images were collected on a Nikon TE2000e microscope with Plan Apo 10× (0.45 numerical aperture) air objective. For the sperm visualization experiments, multiple planes of focus were imaged so as to capture all sperm. The images in Figures 2 and 3 are overlays of one, two, or three focal planes of the fluorescent channels, with the number chosen in each case to best display all the sperm. In each panel a low-contrast brightfield image, to show outline of tissue, is overlaid with green-channel image to show *protamine-GFP* sperm and red-channel image (magenta) to show SSC (*Send1-nRFP*).

## Supporting Information

Figure S1 **TUNEL assay of SSC ablation after mating.** (A–D) Control female 31 h postmating. (E–H) Experimental female 31 h postmating. (I–L) Experimental female 43 h postmating. Control females are *+/UAS-hid^Ala5^; Send1-nRFP/+; MKRS/+* sisters of *+/UAS-hid^Ala5^; Send1-nRFP/+; Send2-GAL4/+* experimental females. (A), (E), and (I) show brightfield images of lower reproductive tract overlaid with red-channel nRFP images (magenta) and green-channel TUNEL reagent images. Red-channel images are shown separately in (B), (F), and (J), green-channel images are shown separately in (C), (G), and (K), and red- and green-channel images are shown merged in (D), (H), (L). In the merged images, overlap between magenta and green signals appears as white.
(TIF)

Table S1 **Presence and appearance of sperm in spermathecae and seminal receptacle.**
(PDF)

Video S1 **Sperm motility in seminal receptacle of control female.** Control female at 5 d postmating. Note motility of sperm and lack of clumping.
(MOV)

Video S2 **Loss of sperm motility in seminal receptacle of SSC-ablated female.** Experimental female at 5 d postmating. Note reduced sperm motility and clumping of sperm heads.
(MOV)

Video S3 **Live birth of fully developed embryo by an ovoviviparous female.** Movements of the larval mouth hooks and trachea are visible through the eggshell, shortly after release from a female with SSC ablated prior to mating.
(AVI)

## Abbreviations

GFP: green fluorescent protein
RFP: red fluorescent protein
SSC: spermathecal secretory cells
