# Supplementary figures and images for: Sperm-Storage Defects and Live Birth in Drosophila Females Lacking Spermathecal Secretory Cells
Source: PLoS Biol. 2011 Nov 8;9(11):e1001192. doi: 10.1371/journal.pbio.1001192 (PMC3210755; doi:10.1371/journal.pbio.1001192)

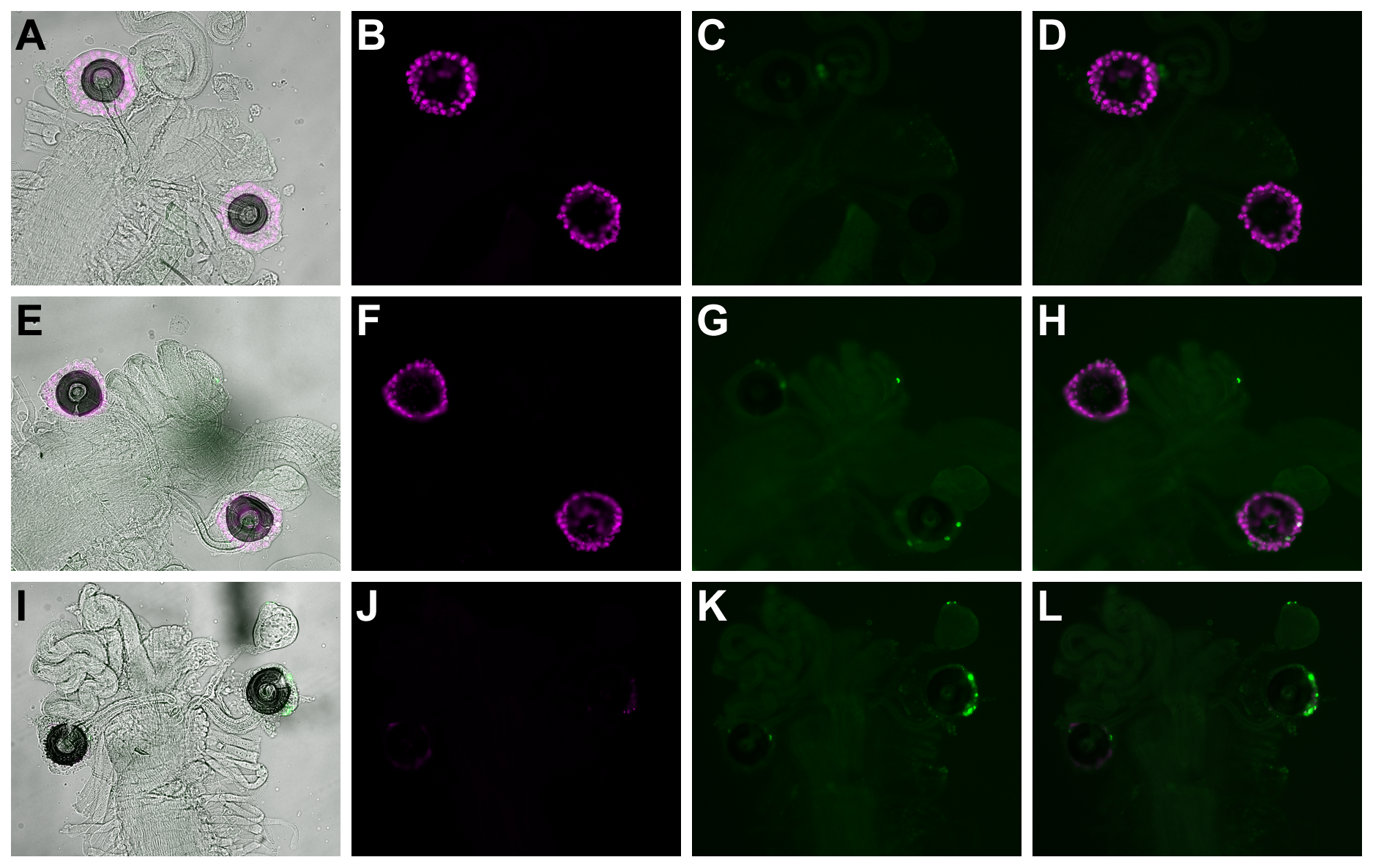

Supplement: Figure S1 — TUNEL assay of SSC ablation after mating. (A–D) Control female 31 h postmating. (E–H) Experimental female 31 h postmating. (I–L) Experimental female 43 h postmating. Control females are +/UAS-hidAla5; Send1-nRFP/+; MKRS/+ sisters of +/UAS-hidAla5; Send1-nRFP/+; Send2-GAL4/+ experimental females. (A), (E), and (I) show brightfield images of lower reproductive tract overlaid with red-channel nRFP images (magenta) and green-channel TUNEL reagent images. Red-channel images are shown separately in (B), (F), and (J), green-channel images are shown separately in (C), (G), and (K), and red- and green-channel images are shown merged in (D), (H), (L). In the merged images, overlap between magenta and green signals appears as white. (TIF) [file pbio.1001192.s001.tif]
