# Supplementary material for: Sperm-Storage Defects and Live Birth in Drosophila Females Lacking Spermathecal Secretory Cells
Source: PLoS Biol. 2011 Nov 8;9(11):e1001192. doi: 10.1371/journal.pbio.1001192 (PMC3210755; doi:10.1371/journal.pbio.1001192)
Supplement: Table S1 — Presence and appearance of sperm in spermathecae and seminal receptacle. (PDF) [file pbio.1001192.s002.pdf]

Table S1. Presence and appearance of sperm in spermathecae and seminal receptacle

		Spermatheca A	Spermatheca B	Seminal receptacle
**1 hour post-mating**				
Control females				
	High	8	7	8
	Low	0	1	0
	Empty	0	0	0
SSC-ablated females				
	High	3*	0	16
	Low	6	1	1
	Empty	8	16	0
**7 hours post-mating**				
Control females				
	High	7	6	7
	Low	0	1	0
	Empty	0	0	0
SSC-ablated females				
	High	0	0	7
	Low	5	1	0
	Empty	2	6	0
**24 hours post-mating**				
Control females				
	High	10	9	12 (2)
	Low	3	3	2 (1)
	Empty	1	2	0
SSC-ablated females				
	High	2	1	11 (2)
	Low	5	2	2 (2)
	Empty	6	10	0
**6 to 8 days post-mating**				
Control females				
	High	7	4	7
	Low	0	3	0
	Empty	0	0	0
SSC-ablated females				
	High	2*	2	4
	Low	7	2	12 (7)
	Empty	7	12	0

Control females are *+/UAS-hid*^{Ala5}*; Sp/+; Send1-nRFP/+* sisters of *+/UAS-hid*^{Ala5}*; CyO, Send1-GAL4/+; Send1-nRFP/+* females with SSC ablated prior to mating. Spermathecae A and B are defined as the ones containing more or less sperm, respectively, within a given female. Amount of sperm in a spermatheca is defined as: High = more than 10 sperm, Low = 1–10 sperm and Empty = 0 sperm. Amount of sperm in a seminal receptacle is defined as: High = sperm throughout all lobes of receptacle, and Low = sperm predominantly localized to one lobe of seminal receptacle. For the spermathecae, each asterisk indicates an individual mosaic female with at least one visible red-fluorescent SSC cell. For the seminal receptacle, the number in parentheses indicates the number in which clumps of sperm were observed.
